# Treated Like Animals: Improving the Lives of the Creatures we Own, Eat and Use

**DOI:** 10.1017/awf.2023.29

**Published:** 2023-03-30

**Authors:** Moira Harris

**Affiliations:** Shrewsbury, UK

Certain books are as famous for their far-reaching effects as for their contents. In 1964, for example, Ruth Harrison’s *Animal Machines* informed the British public about the conditions of intensively farmed animals; the outcry led directly to the publication of the influential *Brambell Report* and focused the attention of the UK Government on a new scientific field, the study of animal welfare. *Animal Machines* was not an academic textbook. It expressed a particular point of view and, as well as raising awareness about the conditions and treatment of animals, it cleverly also appealed to readers concerned about nutrition, food safety and the aesthetics of the countryside.

*Treated Like Animals*, written by Alick Simmons, is a recent volume that aims to educate and inform the non-specialist public. As illustrated by its subtitle, *Improving the Lives of the Creatures We Own, Eat and Use*, the purpose of Simmons’ book is to enlighten readers about the many uses of non-human animals (types of ‘exploitation’, to use Simmons’ terminology) and how readers can (if they choose) act to improve animals’ lives.

As an author, Simmons is well-informed and well-qualified to write about animals. He is a veterinarian of over 30 years’ experience and the UK Government’s former Deputy Chief Veterinary Officer, as well as a naturalist. Accordingly, the book is well-written and full of relevant facts, clearly and truthfully describing a wide range of animal-related practices (many of which readers will find unpalatable). Like *Animal Machines*, it is not an academic book or one aimed at specialists, and its target audience, readers who know little about animal production (or wildlife management or the use of animals in research) will learn much about these practices. It also educates its readership about important concepts in animal welfare, such as behavioural needs, comparative cognition, sentience and the capacity to suffer, as well as lots about the behavioural biology of various species of animals. This is all explained (for the most part) in a logical and accessible way, within a fairly compact volume. There is also some information on important historical milestones like the publication of *Animal Machines*, the subsequent formation of the Brambell Committee and production of the crucially influential *Brambell Report*, and the resultant Five Freedoms and more recent Five Domains animal welfare frameworks.

The book is written from Simmons’ own ethical stance, and he refers to this and the choices he makes as a result frequently. This doesn’t include being a vegetarian, although he describes himself as being “… picky about where my meat comes from and avoid(ing) certain types altogether” and it does include fishing. He believes, according to the Preface, “… that limited and highly specific forms of animal exploitation can be justified” – such as types of animal production that are good for the environment, interventions to protect threatened species and research on human diseases using animals. Later in the book it becomes apparent that the author supports many forms of exploitation – including the consumption of a rather wide range of animal products. Some readers (including those who choose to forego eating meat or adopt a vegan lifestyle as part of their own ethical framework) will certainly find this doesn’t go far enough. He describes veganism as “too rigid” a stance that ignores the benefits of animal use and fails to take into account differences between the best and worst types of use. He also includes advice, such as cautioning against ‘evangelising’ about dietary choices: “Especially at social events. By all means explain, but don’t think you have to convert the world. No one wants a sermon at a party.” Of course, one person’s evangelising is another’s way of sharing important information and some will find this ‘advice’ patronising, preferring to be more vocal – even at parties.

While it is not as fully referenced as an academic textbook, the endnotes provide reference sources to substantiate a lot of the factual information – and these comprise a mixture of scientific journal articles, textbooks, legislation and government reports (i.e. what the academic reader would consider reliable references) as well as, for example, media articles and opinion polls.

Chapter 1 defines what Simmons means by exploitation and also serves as an extended introduction to the rest of the book. In it, he explains why he prefers the term ‘exploitation’ to differentiating practices into those that are benign (‘animal use’) and those that are detrimental to animals (‘abuse’). This is because the terms are subjective and vary between individuals: one person’s use is another’s abuse. Exploitation, in this book, includes everything from the best to the worst impact on animals, from the most minimally interactive (such as observing conserved wildlife) to more significant interventions like eating the meat of animals or wearing their skins, and covers very common interactions that many people are unaware of (because they are hidden from sight) or choose to ignore, like the use of rodenticide poisons for pest control and the killing of one species of animal to facilitate the conservation of another.

Chapter 2 considers why all animals are not treated and regarded in the same way, and the differences in protection and care between closely related species. As it points out, there are odd and anomalous gaps in legislation and differences in the scope of coverage and level of detail between laws that relate to animals – strikingly, between laboratory and wild animals, for example. Some of these oddities are related to societal and cultural norms and our perceptions of the value of different species of animals.

Chapters 3 to 5 are about the main farmed species – grazing animals (cattle and sheep), pigs, poultry and a brief mention of turkeys, ducks and farmed fish. Each chapter describes how the animals are farmed and the impacts on their welfare of aspects including extreme selection for growth or milk/egg production, disease, mutilations like castration and tail-docking, transport and slaughter. Chapter 6 evaluates wildlife and wildlife management, Chapter 7 conservation, Chapter 8 the uses of animals in sport, Chapter 9 pets and Chapter 10 research. In each, the factual and legislative context is explained in a logical and easy-to-understand way.

Chapter 11 is described as ‘A Personal Ethical Framework’ although it doesn’t constitute a framework in the usual sense. In it, the author discusses ethics and morals (and elsewhere in the book he describes the ethical theory of utilitarianism, classifying his own position on animal use as utilitarian). It includes a table listing animal species and groups and his own decisions in relation to these, such as the types of meat he will/will not eat (that the reader may or may not find useful), as well as tips for putting into practice making changes to one’s diet.

Chapter 12, entitled ‘Making Sense of It All’, advocates, among other things, more consistency in our relationship with animals. In it are a number of suggestions, based on the contents of previous chapters, for ways in which our exploitation of animals could be changed. It calls for greater transparency and openness in the use of animals (citing examples of organisations that demonstrate good practice in these respects) and the increased involvement of citizens (including, but not limited to, consumers of animal products) in campaigning for change. It recommends wider involvement of laypersons in making decisions in all areas of animal exploitation. It also suggests – with some justification – that it would be very helpful to stop dividing animals into arbitrary categories, such as ‘farmed’, ‘companion’, and ‘research’ animals, instead concentrating on their experiences and needs. As an example, it makes no difference to a rat whether it is a pet, a lab animal or a ‘pest’, yet its experiences and the rules governing its treatment are currently completely different. While many of Simmons’ ideas and suggestions seem sensible and logical, and will resonate with his readers, others – such as buying more expensive animal products or rearing one’s own chickens – simply will not be available for many, including people living on low incomes or in gardenless apartments.

The book has some limitations. My uncorrected, advance reading copy had a few anomalies and errors, which I would expect to have been corrected in the published version. The glossary and list of abbreviations is helpful but not exhaustive, meaning that some looking-up of technical terms will be needed. It is also slightly out-of-date in its reference to certain legislation since, at around the same time that the book was completed in the spring of 2022, the Animal Welfare (Sentience) Act 2022 formally recognised cephalopod molluscs and decapod crustaceans (as well as all vertebrate non-human animals) as sentient in England, Wales, Scotland and Northern Ireland. The book does refer to the ‘upcoming’ Animal Sentience Bill, both in relation to the omission of invertebrates from the Animal Welfare Act 2006 and the fact that, as the UK has left the European Union, the Lisbon Treaty recognising animals as sentient no longer applies. In terms of geographical remit, the book is very much UK-centric in its references to practices and legislation (although some chapters make reference to other countries). Therefore, readers outside the UK may find it less relevant and interesting.

There is also an unexplained omission of one large category of animal-keeping. Curiously, even though most other uses of and interactions with animals are discussed quite fully, there is virtually no mention of the welfare of animals kept in zoos and wildlife parks, a widespread and controversial practice that (despite some education and conservation benefits) many find unacceptable. Why isn’t zoo animal welfare included? The many species of animals kept in zoos are the subject of large amounts of published research, so it’s not clear why they have been omitted.

Simmons’ stance on sentience is rather conservative. He supports the presumption that (most) mammals are sentient but is more reticent about other animal groups: “While there is consensus that most mammals are sentient, or at least they are given the benefit of the doubt, the extent to which the same view is applied to other groups… such as birds, fish, and reptiles remains the subject of vigorous debate.” Many would contend it is highly likely that sentience extends far beyond mammals to birds, fish and reptiles. Indeed, the new Animal Welfare (Sentience) Act formally recognises that cephalopod molluscs – such as octopus and squid – and decapod crustaceans, including lobster and crab, are sentient.

So, could 2023’s *Treated Like Animals* have a similarly far-reaching impact to that of 1964’s *Animal Machines*? Recently, I asked a group of postgraduate students whether a contemporary book could have such an influence. They suggested that – in an age of abundant media and social media channels – it was unlikely. However, the impact of Alick Simmons’ book remains to be seen; it will educate and inform its readers, and education (along with calls to action) has the potential effect change. Perhaps in sixty years we’ll look back on *Treated Like Animals* as helping to initiate a new era of openness, transparency and citizen engagement in all forms of the exploitation of animals.

